# Impact of PET reconstruction protocols on quantification of lesions that fulfil the PERCIST lesion inclusion criteria

**DOI:** 10.1186/s40658-018-0235-6

**Published:** 2018-12-07

**Authors:** Joke Devriese, Laurence Beels, Alex Maes, Christophe Van de Wiele, Hans Pottel

**Affiliations:** 1 Department of Public Health and Primary Care @ Kulak, KU Leuven campus Kulak, Etienne Sabbelaan 53, 8500 Kortrijk, Belgium; 2 Department of Nuclear Medicine, AZ Groeninge, President Kennedylaan 4, 8500 Kortrijk, Belgium

**Keywords:** 18F-FDG PET/CT, Quantitation, Standardized uptake value, Reconstruction protocol

## Abstract

**Background:**

The aim of this study was to compare liver and oncologic lesion standardized uptake values (SUV) obtained through two different reconstruction protocols, GE’s newest clinical lesion detection protocol (Q.Clear) and the EANM Research Ltd (EARL) harmonization protocol, and to assess the clinical relevance of potential differences and possible implications for daily clinical practice using the PERCIST lesional inclusion criteria.

NEMA phantom recovery coefficients (RC) and SUV normalized for lean body mass (LBM), referred to as SUV normalized for LBM (SUL), of liver and lesion volumes of interest were compared between the two reconstruction protocols. Head-to-toe PET/CT examinations and raw data from 64 patients were retrospectively retrieved. PET image reconstruction was carried out twice: once optimized for quantification, complying with EARL accreditation requirements, and once optimized for lesion detection, according to GE’s Q.Clear reconstruction settings.

**Results:**

The two reconstruction protocols showed different NEMA phantom RC values for different sphere sizes. Q.Clear values were always highest and exceeded the EARL accreditation maximum for smaller spheres. Comparison of liver SUL_mean_ showed a statistically significant but clinically irrelevant difference between both protocols. Comparison of lesion SUL_peak_ and SUL_max_ showed a statistically significant, and clinically relevant, difference of 1.64 and 4.57, respectively.

**Conclusions:**

For treatment response assessment using PERCIST criteria, the harmonization reconstruction protocol should be used as the lesion detection reconstruction protocol using resolution recovery systematically overestimates true SUL values.

## Background

In oncological ^18^F-FDG PET/CT imaging, quantitative analysis is gaining popularity [1, 2]. Standardized uptake value (SUV) is a semiquantitative parameter and serves as a measure for glucose uptake and thus metabolic cell activity for target organs or volumes of interest (VOIs). It facilitates tumour detection, staging, and therapy follow-up, and in the context of multicentre studies, it is essential that SUVs are accurate and reproducible [1–3]. There are several physical, technical, and physiological factors that introduce variability and influence SUV [4]. In the past years, several guidelines have been published and updated that make recommendations about the entire scan and analysis process, e.g., patient preparation, SUV normalization, and VOI positioning [1, 5–7].

Besides these guidelines, several FDG-PET/CT accreditation programmes exist, a.o. the American College of Radiology (ACR) accreditation program [8]. In Europe, the European Association of Nuclear Medicine (EANM) launched the EANM Research Ltd (EARL) accreditation programme. The primary goal of EARL is to harmonize FDG-PET/CT results in multicentre studies by reducing inter- and intra-centre variability in SUVs. The EARL guidelines provide a PET/CT system validation and quality control programme [1, 9]. The image reconstruction procedure, the entire process from raw data to PET images, contains several settings and procedures that may differ between vendors. Reconstruction following manufacturers’ recommendations (e.g. GE’s Q.Clear protocol) is optimized for lesion detection, while EARL reconstruction recommendations are optimized for harmonized quantitative analyses. These two reconstruction protocols do not necessarily go hand in hand in clinical practice. Proposed solutions include performing both reconstructions independently, using one for visual (diagnostic) assessment and the other for quantitative assessment [10]. Another possible solution is to apply two PET reconstruction protocols in a single image processing procedure [11]. The latter proposed solution was developed into proprietary software and has been validated in a multicentre study [12].

The aim of this study was to compare liver and oncologic lesion SUV values obtained through two different reconstruction protocols: GE’s newest clinical lesion detection protocol (Q.Clear) and the EARL harmonization protocol, using the PERCIST lesional inclusion criteria. We will assess the clinical relevance of potential differences between both methods and the possible implications in routine clinical practice.

## Methods

### Phantom preparation

To evaluate quantitative PET accuracy, a NEMA NU 2 IEC body phantom with six spheres was used with sphere diameters of 10, 13, 17, 22, 28, and 37 mm. The phantom was filled with ^18^F-FDG according to EARL guidelines [1].

### Patients

The raw data from 64 PET/CT examinations covering head to toe acquired for clinical indications over a period of 13 months were retrospectively retrieved. Patients fasted for at least 6 h and were only injected with FDG and scanned when serum glucose levels were lower than 200 mg/dl. FDG doses adjusted on a linear basis for patients’ body weights were administered intravenously. Patients breathed freely during acquisition. Studies using retrospectively collected and anonymized data do not require institutional review board approval according to the Belgian law.

### PET/CT acquisition and reconstruction

All examinations were performed on a GE Discovery 710 system (GE Medical Solutions, Waukesha, WI, USA) with software version 52.00. CT scan parameters were 120 kV, 80–180 mA (auto), 700 mm field of view (FOV), 1.25 mm slice thickness, and 512 × 512 matrix size. The raw PET data were reconstructed twice.

On the one hand, the data were reconstructed with reconstruction settings optimized for quantification, using the ordered subset expectation maximization (OSEM) reconstruction algorithm. This reconstruction protocol meets the EARL requirements and is further on referred to as the EARL protocol. Details concerning the EARL PET acquisition and reconstruction are presented in Table 1.

**Table 1:** EARL PET acquisition and reconstruction parameters

	Patients	NEMA phantom
Time per bed position	1.5 min	10 min
Reconstruction	OSEM + TOF	
Iterations/subsets	3/24	
Post filter	9 mm	
Matrix size	256 × 256	
Pixel spacing	2.73	
Slice thickness	3.27 mm	

*OSEM* ordered subset expectation maximization, *TOF* time of flight

On the other hand, the data were reconstructed with reconstruction settings optimized for lesion detection, using the Q.Clear reconstruction algorithm (GE Medical Solutions, Waukesha, WI, USA).

### Optimization of *β*, penalization factor

The Q.Clear protocol is a Bayesian penalized likelihood reconstruction algorithm which incorporates a penalty factor to control noise [13]. It includes time of flight (TOF) and point spread function (PSF), taking into account resolution-degrading effects such as positron range, photon non-collinearity, and detector-related effects including crystal widths, inter-crystal scattering, and inter-crystal penetration (depth of interaction effects). The use of the relative difference penalty and the modified block sequential regularized expectation maximization (BSREM) allow full convergence. Only one user input parameter is necessary: *β*, penalization factor [14].

To optimize the Q.Clear reconstruction settings, five different *β* penalization factors were used to reconstruct the NEMA NU 2 IEC body phantom. The RC mean and RC max were calculated for the different spheres. Furthermore, image quality (lesion detectability and noise minimization) with *β* penalization factor 350 and 400 was assessed by 2 experienced nuclear medicine physicians for 20 patients.

### Image analysis

The phantom data were analyzed using the EANM QC tool version 15/8/2011. The recovery coefficient (RC) equals the ratio of measured radioactivity concentration to true radioactivity concentration.

The patient data were analyzed using PMOD software (PMOD Technologies Ltd, Zurich, Switzerland) with software version 3.6. Liver and lesion VOIs were placed according to the recommendations by Wahl et al. [6]. A 3-cm diameter sphere was placed in the right hepatic lobe. All patients’ livers were healthy. Mean SUV (SUV_mean_) was determined for each liver VOI. Up to five lesions per patient were selected, maximum two lesions per organ, with the most intense FDG uptake. Max and peak SUV were determined for each lesion. SUV_max_ corresponds to the maximal recorded SUV within the lesion. SUV_peak_ is determined by placing a 1-mL VOI in the lesion, positioned to obtain the highest possible mean SUV within that VOI. SUV_peak_ is the mean SUV of that VOI. SUV is normalized for lean body mass (LBM; Janmahasatian et al. [15]) and is further on referred to as SUV normalized for LBM (SUL).

### Statistical analysis

Descriptive statistics (mean (SD) or median (IQR) where appropriate) and boxplots were used to present the characteristics of the samples. Bland-Altman analyses were used to compare SUL differences between the two reconstruction protocols. All Bland-Altman plots display absolute SUL values and differences unless stated otherwise. All calculations and analyses were performed with Microsoft Excel 2013 (Microsoft Corp., Redmond, WA, USA) and GraphPad Prism version 6.07 for Windows (GraphPad Software, La Jolla, CA, USA).

## Results

### Optimization of *β*, penalization factor

To optimize the Q.Clear reconstruction, the *β* penalization factor was varied from 200–600. The RCs for the different *β* factors can be found in Table 2.

**Table 2:** Optimization of Q.Clear *β* penalization factor

Sphere diameter (mm)	RC mean					RC max				
	*β* 200	*β* 350	*β* 400	*β* 450	*β* 600	*β* 200	*β* 350	*β* 400	*β* 450	*β* 600
10	0.97	0.83	0.78	0.76	0.66	1.42	1.18	1.12	1.06	0.93
13	1.01	1.01	0.99	0.98	0.93	1.39	1.32	1.30	1.28	1.22
17	0.89	0.88	0.86	0.87	0.86	1.13	1.11	1.11	1.12	1.12
22	0.92	0.89	0.89	0.88	0.88	1.26	1.17	1.15	1.14	1.10
28	0.94	0.91	0.91	0.91	0.90	1.16	1.11	1.09	1.09	1.07
37	0.94	0.93	0.92	0.92	0.92	1.18	1.12	1.11	1.11	1.09

*RC* recovery coefficient

The RCs (mean) for the investigated *β* penalization factors exceeded all EARL accreditation maximum values. The RCs (max) for *β* 200 exceeded all EARL accreditation maximum values, while for the *β* 350–600, RCs (max) exceeded EARL accreditation maximum only for spheres of 22 mm diameter and smaller.

The physicians preferred the image quality, both lesion detection and noise minimization, with the Q.Clear *β* penalization factor set to 400. Therefore, *β* penalization factor 400 was used to reconstruct the patient data.

### Phantom recovery coefficients

**Fig. 1:**
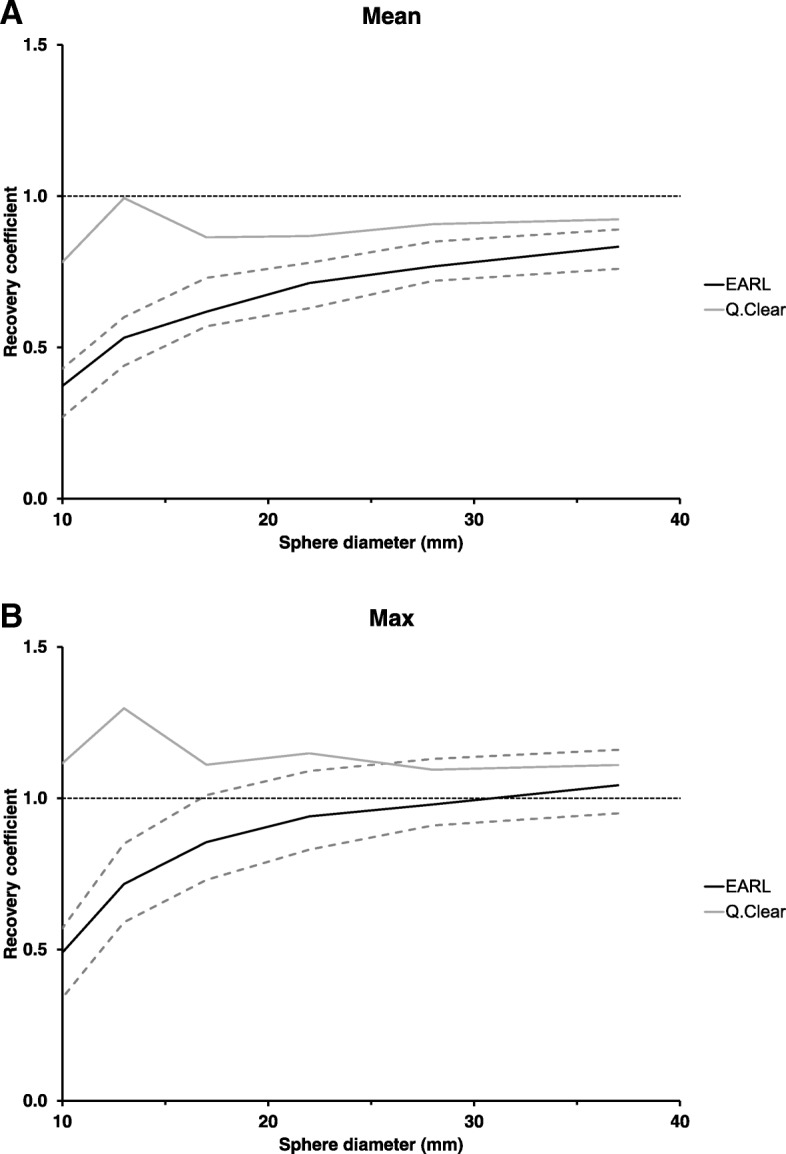
NEMA phantom recovery coefficients for the mean (**a**) and maximum (**b**) pixel values (dashed lines: EARL accreditation minimum and maximum values)

The RCs for the mean and maximum pixel values are presented in Fig. 1. The EARL values completely lied within the EARL accreditation minimum and maximum values, while the Q.Clear values were always higher than the EARL values.

### Patient characteristics

The 64 patients included in this study were all Caucasian, 17 men and 47 women. Mean (SD) patient age was 63.1 (15.9) years, and mean (SD) BW was 73.9 (16.7) kg. Mean (SD) FDG uptake time was 61.7 (3.9) min, all within 50 to 70 min.

For every lesion, minimal metabolically measurable tumour activity (PERCIST) [6] was assessed for both reconstruction protocols separately. Of 64 patients, 19 had one or more lesions that fit this criterion and were thus deemed quantitatively interpretable. Of all 47 lesions, 15 fit the criterion after both reconstructions, 18 fit the criterion only after Q.Clear reconstruction, and 14 lesions did not fit the criterion after either reconstruction. The first two groups, 33 lesions, were included in the following analyses and are distinctively represented in all figures: ● for lesions deemed quantitatively interpretable after both reconstructions, ○ after Q.Clear reconstruction only. Lesions were mainly from melanoma patients (19), but also bone metastases of unknown origin (7), adenocarcinoma (4), renal cell carcinoma (2), and sarcoma (1). Diameters of 25 lesions were measured on CT, and 8 were considered unmeasurable due to diffuse lesion boundaries (e.g., bone lesions). Median (IQR) lesion diameter was 18 (17.5) mm.

### Liver SUL_mean_

**Fig. 2:**
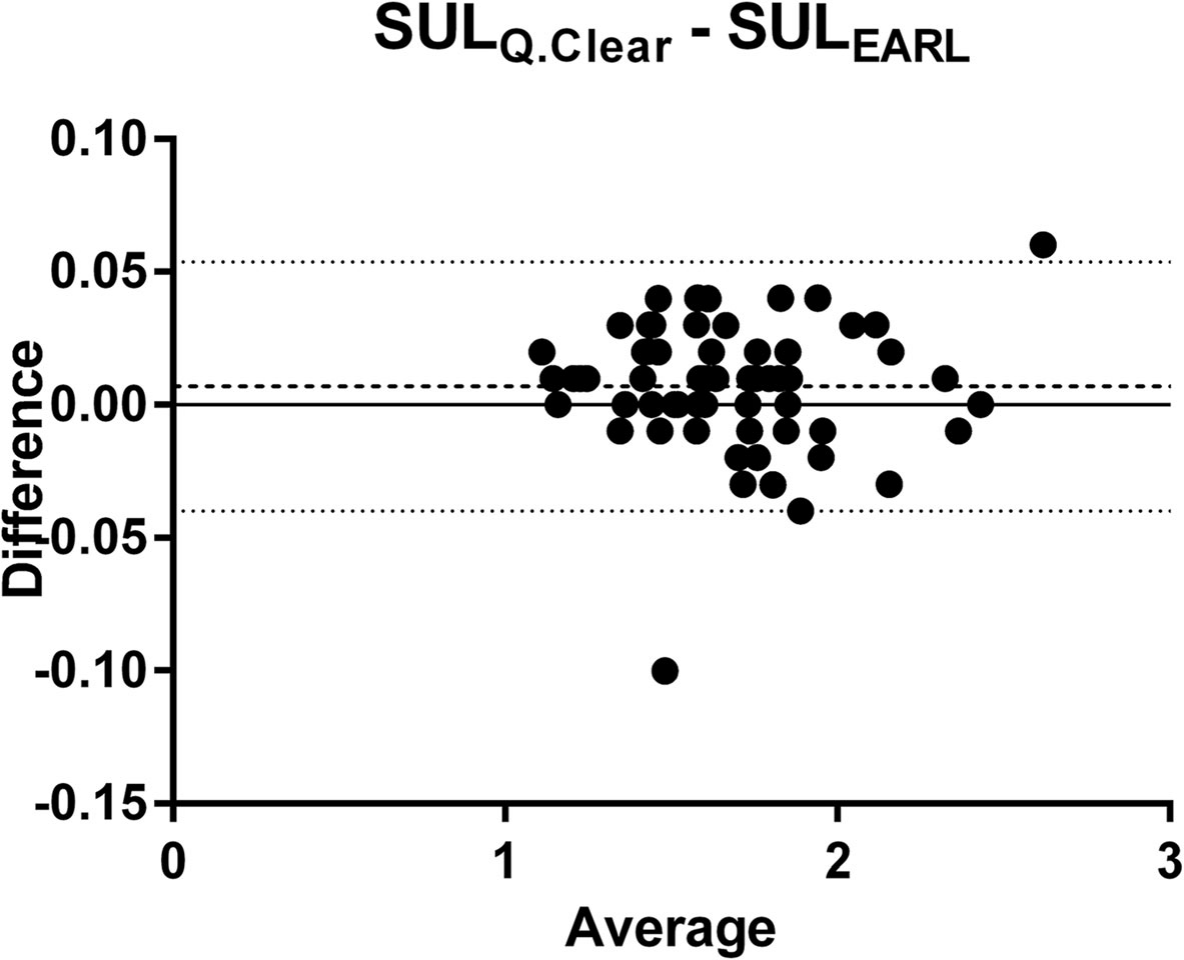
Bland-Altman plot of liver SUL_mean_ comparison between two reconstruction protocols (dashed line: mean difference, dotted lines: LOA)

Mean (SD) liver SUL_mean_ for all 64 patients was 1.682 (0.312) for the Q.Clear protocol and 1.675 (0.312) for the EARL protocol. A pairwise comparison was made via Bland-Altman analysis, displayed in Fig. 2. A statistically significant difference of 0.007, with a 95% confidence interval (CI) ranging from 0.002 to 0.012, was observed. The 95% limits of agreement (LOA) are [− 0.040, 0.054].

### Lesion SUL_peak_ and SUL_max_

**Fig. 3:**
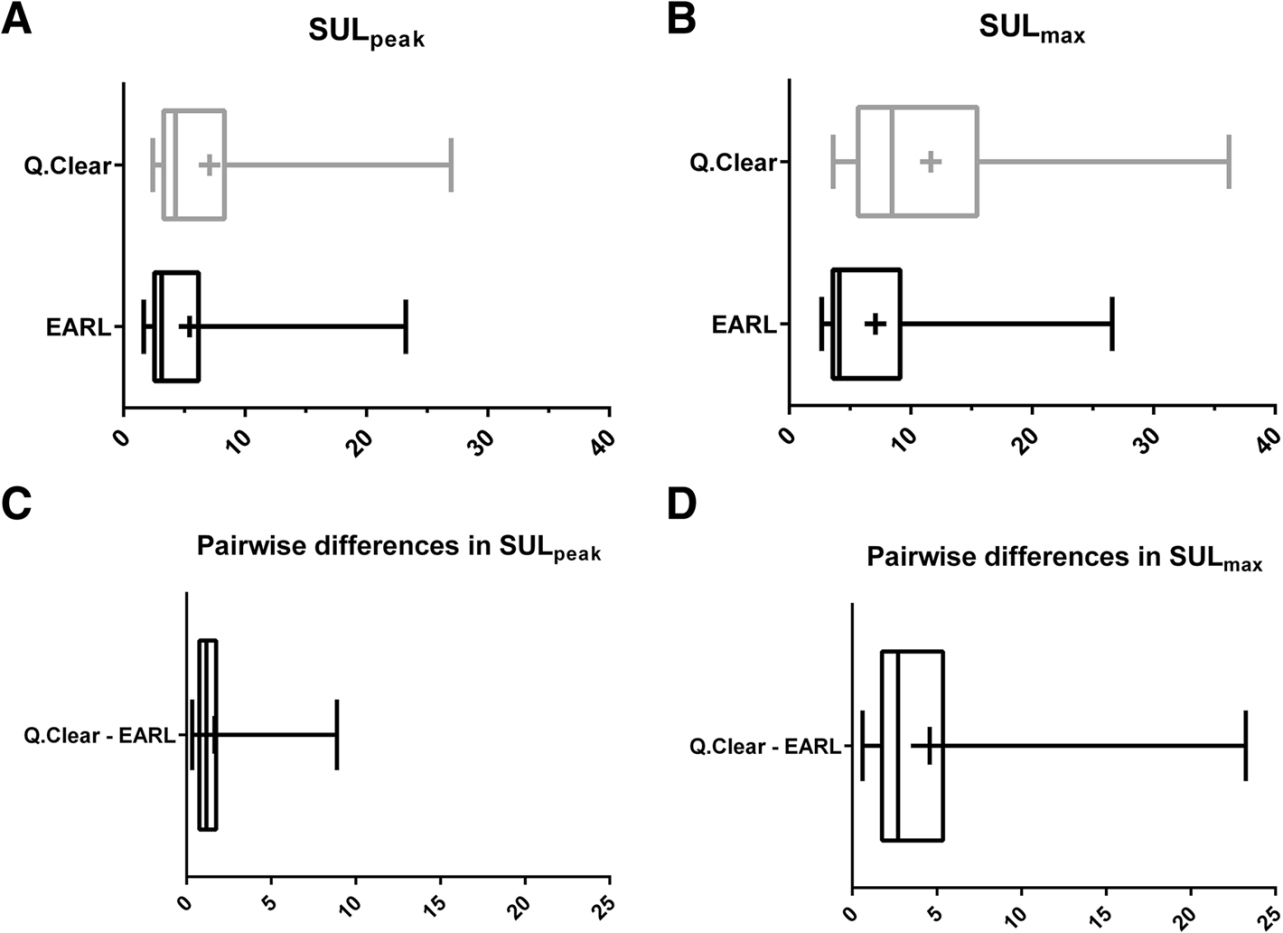
Boxplots showing the distribution of lesion SUL_peak_ (**a**) and SUL_max_ (**b**) and pairwise differences (**c**, **d**) between the two reconstruction protocols

Descriptive statistics and distribution of lesion SUL for both protocols and pairwise SUL differences are presented in Table 3 and Fig. 3.

**Table 3:** Median (IQR) of lesion SUL_peak_ and SUL_max_ for the Q.Clear and EARL protocol, and Bland-Altman comparison details

	Q.Clear	EARL	Pairwise difference [95% CI]	LOA 95%
SUL_peak_	4.27 (4.99)	3.14 (3.63)	1.64 [1.13, 2.15]	[-1.77, 5.05]
SUL_max_	8.46 (9.80)	4.13 (5.51)	4.57 [3.13, 6.02]	[-5.02, 14.17]

**Fig. 4:**
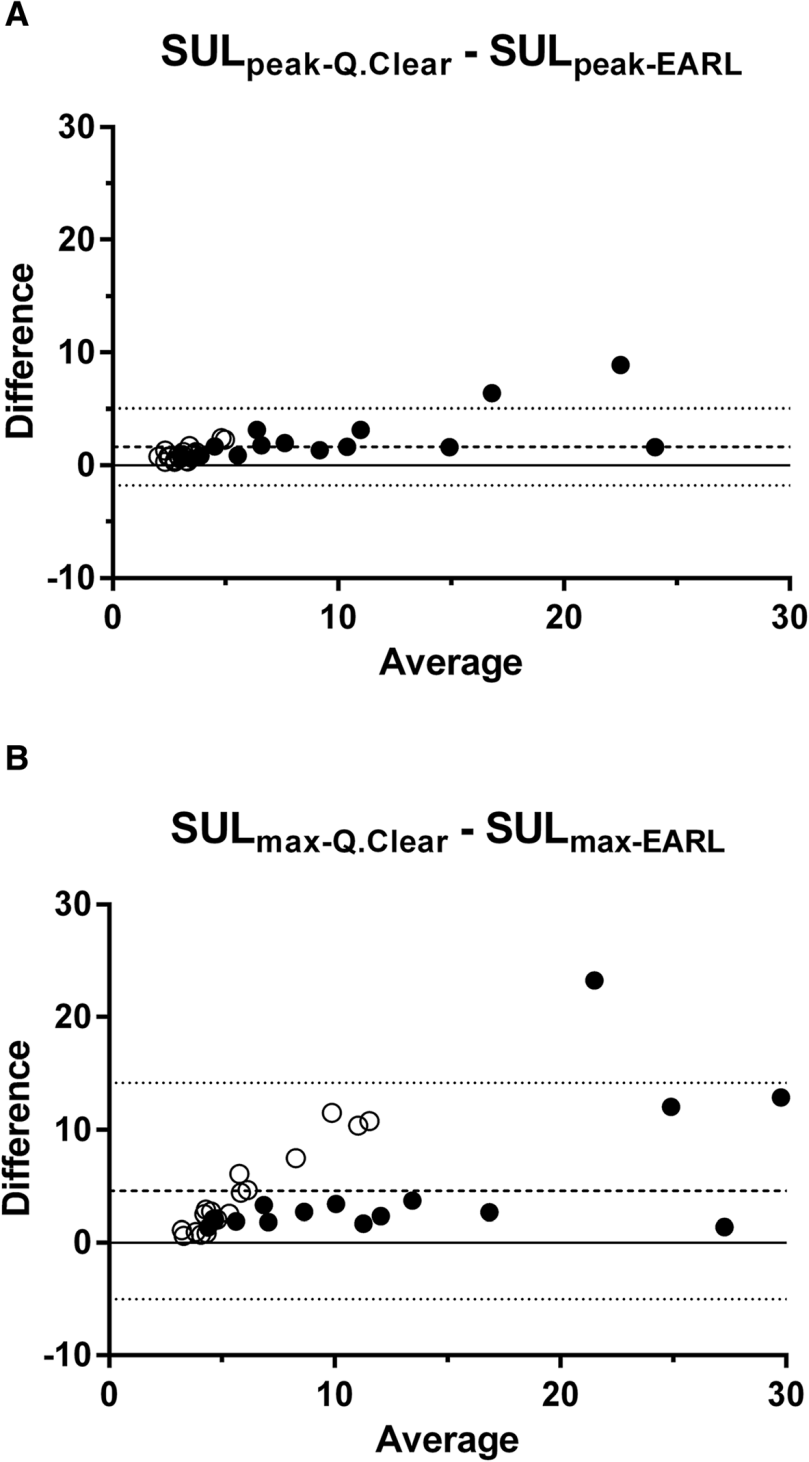
Bland-Altman plots of lesion SUL_peak_ (**a**) and SUL_max_ (**b**) comparison between two reconstruction protocols (dashed line: mean difference, dotted lines: LOA, ●: lesions deemed quantitatively interpretable after both reconstructions, and ○: after Q.Clear reconstruction only)

Bland-Altman plots (difference versus average) are presented in Fig. 4. Bias and LOAs are presented in Table 3.

**Fig. 5:**
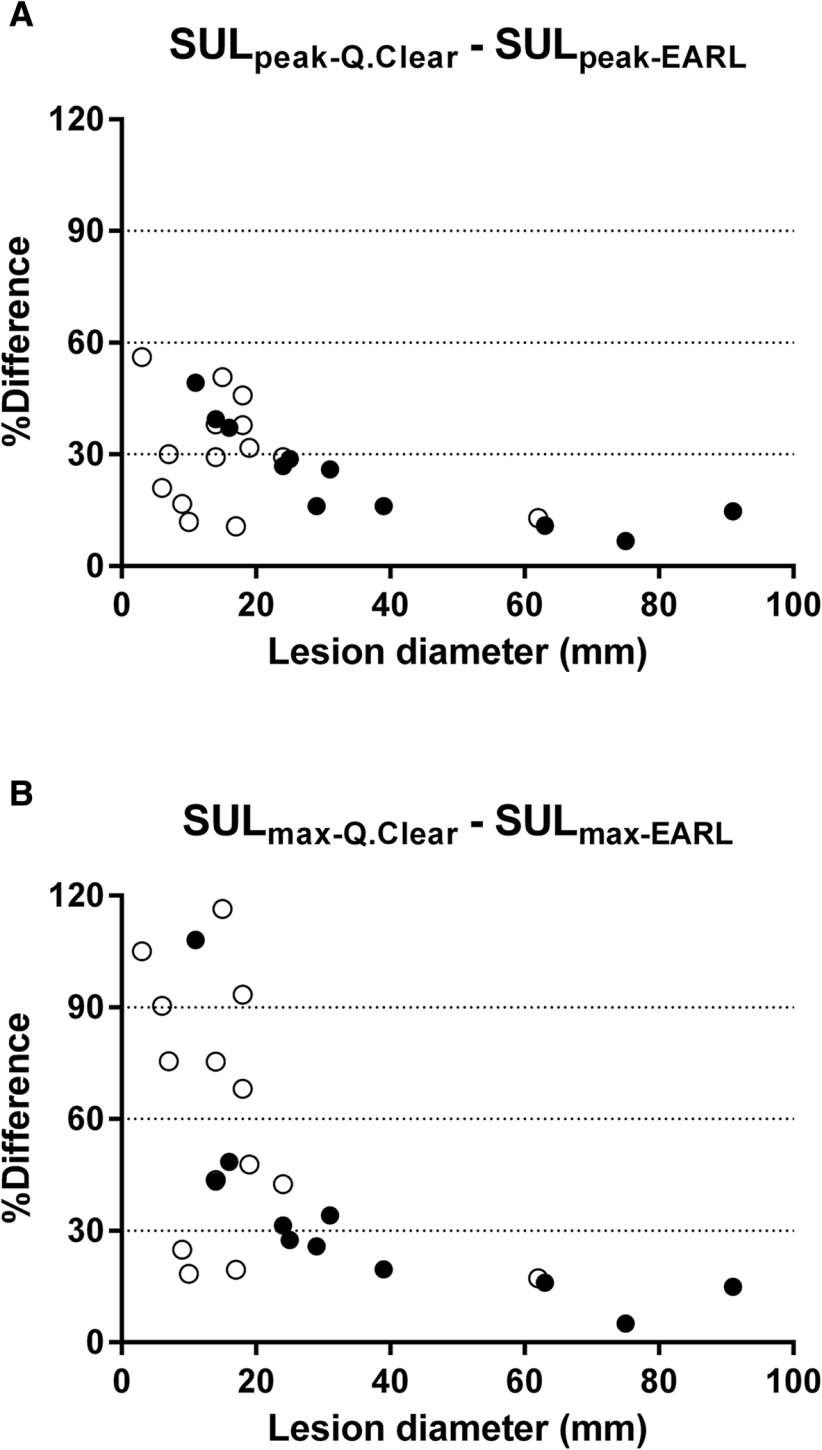
Scatterplots of differences in SUL_peak_ (**a**) and SUL_max_ (**b**) between the two reconstruction protocols vs. lesion diameter (●: lesions deemed quantitatively interpretable after both reconstructions, and ○: after Q.Clear reconstruction only)

Differences in SUL_peak_ and SUL_max_ between the two reconstruction protocols were assessed and plotted against lesion diameters (Fig. 5).

## Discussion

The phantom data analysis shows that RCs lie between the EARL minimum and maximum values after EARL reconstruction for all sphere sizes. After Q.Clear reconstruction, max pixel RCs lie between the EARL minimum and maximum values for the larger spheres but RCs exceed the EARL maximum values for spheres of 22 mm diameter and smaller and mean pixel RCs consistently exceed the EARL maximum values. Q.Clear RCs are higher than EARL RCs for all sphere diameters, which is beneficial for lesion detection. From a quantitative point of view, RCs should be as close to 1 as possible for all spheres. EARL RCs decrease with decreasing sphere diameter, attributable to the partial volume effect [16]. Q.Clear RCs are higher than 1 for all sphere sizes. This was also observed in other studies using PSF, and a relation was seen between overshoot and ROI size and between overshoot and signal-to-background ratio [17–19]. Discontinuities such as sharp changes in image contrast, when approximated by a Fourier series, will be truncated with an overshoot at the border of discontinuity. This phenomenon, first described by Wilbraham in 1848 [20] and later on rediscovered by Gibbs in 1899 [21], is currently termed the Gibbs effect. Thus, when confronted with increasingly small FDG-avid lesions in PET imaging, the Gibbs effect will manifest itself in the form of a visible dip at the lesion centre due to enhanced edges. With sufficiently small lesions, e.g., the smallest spheres included in the phantom study, this will lead to overshoot and thus overestimation of the real SUV value due to merging of the edge artefacts, explaining the RC > 1 and thereby challenging accuracy in quantification of small uptake regions.

The Gibbs phenomenon not only impacts SUV values but also affects volumetric PET parameters such as metabolic tumour volume (MTV), determined through region growing based on percentage SUV thresholds. Several authors have shown an advantage of using MTV for therapy outcome prediction in addition to or even instead of SUV [22–25]. Physical size of a lesion is difficult to derive from PET images due to spill-out and partial volume effects [16, 26]. Without a doubt, it is essential that SUVs are accurate and reproducible in order to determine reliable MTV and assess treatment response. Consider the following example, well-explained by Munk et al. [26] and applied to our phantom data. Assume a patient has a small tumour lesion of 17 mm diameter, and after 2 cycles of a 6-cycle chemotherapy treatment, the tumour shrinks to a 13-mm remnant without any change in physiology. The tumour volume has been reduced by 24%, indicating response to treatment and possibility that the patient will be cured after the remaining chemotherapy cycles. However, if we consider measured radioactivity concentration in our phantom, we see that after Q.Clear reconstruction the 13-mm RC is 15% higher than the 17-mm RC, meaning that we would observe a 15% increase in SUL_max_ in our patient lesion and rather be inclined to conclude disease progression. This discrepancy in treatment response interpretation is caused by PSF artefacts and proves the need for further image reconstruction optimization and PSF artefact suppression when images are used for quantitative treatment response monitoring [26].

In the 64 examined patients, we found a statistically significant difference of 0.007 (0.42%) of healthy liver SUL_mean_ between the two reconstruction protocols. LOA ranged from − 0.040 to 0.054, corresponding to a 5.60% difference between the upper and lower limit. These differences were considered clinically irrelevant, because beneficial therapy response is often associated with a 30% decrease in SUV [6, 27]. This means that the choice of reconstruction protocol does not have an impact on quantification of the liver as reference tissue and that they could be used interchangeably.

For every single lesion, higher SUL_peak_ and SUL_max_ values were obtained after Q.Clear reconstruction than after EARL reconstruction. Larger differences between SUL from the two different protocols are observed for larger SUL values (heteroscedasticity). The mean difference for SUL_peak_ (1.64) corresponds to a % difference of 44.3%, and the mean difference for SUL_max_ (4.57) corresponds to a % difference of 72.4%. Both mean differences are statistically significantly different from zero and clinically (very) relevant. When comparing SUL differences between reconstruction protocols to lesion diameter, we clearly see the following trend. Larger % differences of SUL between reconstructions are observed in smaller lesions, while % differences are lower than 30% for larger lesions. This is the same observation as that made in the NEMA phantom and can be explained by the Gibbs artefact. Differences are also more pronounced in SUL_max_ than in SUL_peak_, as may be anticipated related to VOI size. The single voxel that makes up SUL_max_ is sensitive to noise. Potential outliers are obscured by surrounding voxels (1-ml sphere) when assessing SUL_peak_, explaining why differences between the two PET reconstruction protocols are more extreme in SUL_max_ values.

Several studies have shown an increase in magnitude of quantitative parameters after Q.Clear reconstruction compared with OSEM reconstruction [28–31]. Lasnon et al. [32] compared PSF reconstructed to non-PSF reconstructed OSEM PET scans in non-small cell lung cancer lesions. They reported that the use of PSF increases SUV_max_ by 48% and SUV_mean_ by 28%. They also found an improvement in sensitivity and negative predictive values when using PSF. Akamatsu et al. [33] reported an increase in SUV after use of PSF, TOF, and PSF+TOF compared to conventional OSEM. The authors state that this improves small-lesion detectability, but the accuracy of quantitative measurements is influenced. Brendle et al. [34] found a significant increase of SUV after addition of PSF to the image reconstruction. Bellevre et al. [35] state that the use of PSF increases spatial resolution, thereby improving lesion detectability. When compared to the results obtained in the aforementioned studies using commercially available software provided by SIEMENS, higher SUV_max_ values were obtained in the series presented using resolution recovery software (Q.Clear) provided by GE (average ranging from 3 to 66% for SIEMENS versus 78% in our series including lesions that comply with PERCIST criteria). In this regard, recent data by Armstrong et al. [36] show that notable differences are observed in terms of standardized uptake value recovery when using SIEMENS or GE software and that harmonization techniques will be mandatory when for instance considering multicentre studies using different equipment and resolution modelling software. Furthermore, as opposed to the previously mentioned studies using SIEMENS software, in this series only minimal metabolically active lesions as defined by PERCIST criteria were included, thus potentially including lesions with ab initio high tumour-to-background ratio which may also in part explain the higher SUV_max_ and SUV_peak_ values found using the GE resolution recovery in our series.

Overall, our study supports the statement that harmonizing quantification and optimal lesion detection do not necessarily go hand in hand and are partially vendor-dependent [9]. Despite all recent progress and efforts, there are still a lot of questions remaining. It seems advisable to apply the lesion detection protocol in diagnostic clinical contexts for individual patients. However, it also seems advisable to select the EARL protocol in multicentre studies and individual therapy response monitoring, in order to reliably compare SUL among patients, scanners, and centres. However, it is clear that PET exams of individual patients should always be examined using the best possible lesion detection protocol. One may wonder whether centres participating in multicentre studies should always carry out two reconstruction protocols. Can lesion detection protocols be implemented in the EARL accreditation program? Or would it be possible to harmonize quantification and lesion detectability without trade-offs?

A limitation in this study is the low number of patients and lesions included. This study could also be extended to other centres using the same PET system for reproducibility assessments. In this study, no extensive comparison of different (OSEM) reconstruction settings was performed. The purpose of this study, however, was to assess differences in quantification between PET reconstruction protocols that are clinically available at the time being.

## Conclusions

For treatment response assessment using PERCIST criteria, the harmonization reconstruction protocol should be used as the lesion detection reconstruction protocol using resolution recovery systematically overestimates true SUL values.

## Abbreviations

BSREM: Block sequential regularized expectation maximization
CI: Confidence interval
EANM: European Association of Nuclear Medicine
EARL: EANM Research Ltd
FOV: Field of view
LBM: Lean body mass
LOA: Limits of agreement
MTV: Metabolic tumour volume
OSEM: Ordered subset expectation maximization
PSF: Point spread function
RC: Recovery coefficient
SUL: SUV normalized for LBM
SUV: Standardized uptake value
TOF: Time of flight
VOI: Volume of interest
